# Editorial: Plant-based drugs: the potential novel therapeutic intervention against cancer stemness and metastasis

**DOI:** 10.3389/fphar.2023.1283694

**Published:** 2023-10-06

**Authors:** Vijayasteltar B. Liju, Sunil Martin, Lekshmi R. Nath, Gopa Kumar Gopinadhan Nair, Hamsa Thayele Purayil

**Affiliations:** ^1^ The Shraga Segal Department of Microbiology, Immunology and Genetics, Faculty of Health Sciences, Ben-Gurion University of the Negev, Beer-Sheva, Israel; ^2^ Laboratory of Synthetic Immunology, Cancer Immunotherapy Program, Division of Cancer Research, Rajiv Gandhi Center for Biotechnology, Thiruvananthapuram, Kerala, India; ^3^ Department of Pharmacognosy, Amrita School of Pharmacy, Amrita Vishwa Vidyapeetham, Kochi Campus, Kochi, Kerala, India; ^4^ Department of Ophthalmology, Keck School of Medicine, USC Roski Eye Institute, University of Southern California, Los Angeles, CA, United States; ^5^ Clinical Pharmacology and Bioanalysis, Princeton, NJ, United States

**Keywords:** phytomedicine, cancer stem cells, metastasis, drug resistant, therapeutic, tumor recurrence

Cancer is the second most common fatal disease leading to death worldwide. Metastatic growth in distant organs is the main reason for increased cancer mortality (Steinbichler et al., 2020). Cancer stem cells (CSCs) play a vital role in each step of the metastatic cascade, from cancer cell invasion into blood vessels, survival in the bloodstream, attachment and extravasation to colonization of the host organ and subsequent formation of macro and micro metastases (Gupta et al., 2021). CSCs are a small subpopulation of cells in tumors with self-renewal and differentiation capabilities that contribute to tumor growth, recurrence and metastatic spread. No successful therapies have been developed that target cancer stem cells to prevent metastasis. Currently available cancer treatment approaches including surgical resection, radiotherapy and chemotherapy are not sufficient to target CSCs (Yang et al., 2020). Therefore, we need to focus more on CSC as a major cause of cancer metastasis and compile the latest research to design future studies targeting CSC. Our Research Topic aims to explore current and future strategies for CSC targeted cancer therapies to prevent cancer metastasis in a way that is promising for patients. This editorial aims to provide a brief overview of the current state of research in this area and to highlight the promising aspects and challenges. This Research Topic comprises of five articles, including two original research articles, two reviews and one systemic review.

Cancer stem cells promote metastasis through multiple signaling pathways such as Wnt, Notch, Hh, NF-κB, JAK-STAT and TGFβ/SMAD which can enhance the stemness properties of cancer cells and lead to metastasis (Yang et al., 2020). It is challenging to target cancer stem cells as a population in a cancerous tumor. Therefore, developing therapies targeting cancer stem cells could open up new ways to treat metastatic cancer by disabling these signaling pathways. Plant-derived bioactive compounds gained considerable attention as potential new therapeutic interventions against cancer stem cells and metastases (Gupta et al., 2021). Metabolites derived from various plants have shown promising anticancer properties in preclinical studies. These metabolites can act on multiple signaling pathways involved in cancer stem cell formation, self-renewal, stem cell differentiation and apoptosis in cancer cells (Yang et al., 2020). By targeting CSCs, herbal medicines can potentially overcome treatment resistance and reduce the risk of tumor recurrence and metastasis. Several plant metabolites such as curcumin from turmeric, resveratrol from grapes, quercetin from fruits and vegetables and epigallocatechin gallate from green tea have been extensively studied for their anticancer effects on CSCs and metastases in various *in vivo* mouse models targeting cancer stem cells and their signaling pathways (Ramasamy et al., 2015; Wang et al., 2018; Namiki et al., 2020; Qin et al., 2020; Liao et al., 2023). However, the low bioavailability of plant metabolites as therapeutic agents is a significant limitation. Nevertheless, modifications by structural analogues and special formulations are a promising combination therapy in cancer treatment with chemotherapy and radiotherapy (Hegde et al., 2023). This combination helps to counteract metastasis and is thus an advance in pharmacological and therapeutic terms. Recent studies, including a clinical study of curcumin in patients with invasive breast cancer (NCT03980509) and clinical trials of resveratrol against gastric cancer, illustrate the therapeutic importance of phytometabolites (Honari et al., 2019; Howells et al., 2019; Ostwal et al., 2022). Most of these studies relate to a late recurrence stage with a probable accumulation of CSCs, which offers the opportunity to investigate the effects of plant metabolites on CSCs.

The exploration of plant derived drugs as potential therapeutic interventions against cancer stem cells and metastasis is an exciting area of research that complement existing cancer therapies and improve patient outcomes. Yuan et al. demonstrate the ability of plant metabolite triptolide to suppress glioblastoma tumor. Triptolide transcriptionally suppresses the expression of PROX1 in glioblastoma cells. This metabolite inhibits glioblastoma cell growth, apoptosis, proliferation, migration and invasion, thereby reducing the metastatic potential of glioblastoma. A few clinical studies have been carried out to evaluate their safety and efficacy focusing on phytomedicines targeting drug resistant CSCs and cancer cells. The review article by Zhang et al. illustrate on many of the active ingredients in traditional Chinese herbal medicine (TCM) which are effective in treating pancreatic cancer. The article focuses on the mode of action of bioactive plant metabolites from TCM against various signaling pathways such as Wnt and Notch, which regulate cancer stem cells in pancreatic cancer. Zhang et al. explain the inhibition of cancer stem cell signaling pathways involved in epithelial to mesenchymal transition, migration, metastasis and apoptosis of pancreatic cancer cells by the plant metabolites.

Targeted therapies for the heterogeneous population of cancer tumors and multidrug resistance associated with cancer stem cells are still not effectively established. The review article by Koklesova et al. describes how nanoparticles specifically designed to target cancer stem cells in tumor, serve as carriers for targeted delivery of plant-based drugs. Several Nano-drugs developed to target and treat the different types of cancer and their subsequent CSCs are discussed. Plant metabolite-based nanoparticles are modified to specifically target cancer stem cells, which helps in reducing the risk of tumor recurrence and the manifestation of metastases. Zhao et al. describe the anti-metastatic effect of thymoquinone in pancreatic cancer and its sensitivity to gemcitabine by regulating collagen. In pancreatic cancer cells, thymoquinone promotes apoptosis, inhibits tumor cell migration, invasion and metastasis, and sensitizes pancreatic cancer cells to gemcitabine. Furthermore, the study shows that thymoquinone stimulates cellular matrix production via TGFβ/Smad pathways.

Studies show that flavonoids have an anticancer effect against various types of cancer. Flavonoids can induce apoptosis and autophagic cell death of breast cancer cells, inhibit cancer proliferation and overcome drug resistance of cancer tumors (Mazurakova et al.). In the systemic review article, Mazurakova et al. detail the effect of flavonoids on breast cancer stem cells to combat therapy resistance and cancer proliferation. The accumulation of breast cancer stem cells (BCSCs) after chemotherapy, prevents further effective treatment of breast cancer patients. Therefore, it is also postulated that eradication of BCSCs in combination with standard cytotoxic drugs and flavonoids specifically targeting CSCs in breast cancer tumor is essential for successful treatment.

This Research Topic provides new data and detailed reviews on plant-derived compounds that target cancer stem cells and inhibit metastasis through various mechanisms of action by modulating self-renewal pathways, suppressing EMT, affecting the tumor microenvironment and disrupting signaling pathways essential for cancer cell migration, invasion and survival. Thus, plant metabolites are promising for developing innovative therapeutic strategies against cancer stem cells and metastasis. Prospective research is inevitable to successfully translate research findings into clinical trials by addressing challenges such as safety, bioavailability, formulation and standardization of herbal medicines. These findings and reviews demonstrate how phytomedicines targeting cancer stem cells significantly reduce metastasis and subsequent mortality. These prospective and innovative findings, if clinically established, are promising and can pave the way to new vistas for cancer therapy.
